# Unconventional guardians of the brain: MAIT cells as emerging players in glioblastoma immunity

**DOI:** 10.3389/fimmu.2026.1896626

**Published:** 2026-08-13

**Authors:** Eleanor M. Eddy, Timothy Patton, Alexander D. Barrow, Alexandra J. Corbett

**Affiliations:** Department of Microbiology and Immunology, Peter Doherty Institute for Infection and Immunity, University of Melbourne, Melbourne, VIC, Australia

**Keywords:** brain, GBM, glioblastoma, immune therapy, MAIT cells

## Abstract

Glioblastomas (GBM) are an aggressive form of brain cancer with no curative treatment options. While T cell therapies have demonstrated substantial success in combatting blood cancers, their application for solid tumours, including GBM, is hampered by the immunosuppressive tumour microenvironment and scarcity of tumour antigen-specific targets. There is an urgent need to better understand the immune response to GBM to enable the development of new therapeutic approaches. Recently, a resident population of mucosal-associated invariant T (MAIT) cells in the brain has been described, which may perform critical immune surveillance functions. Recent research also suggests that MAIT cells play diverse roles in anti-tumour immunity, and there is much interest in exploring the potential of MAIT cells in immune therapy by harnessing their cytotoxic capacity and tissue homing properties. However, findings on the role of MAIT cells in cancer are conflicting, while many basic questions regarding MAIT-tumour cell interactions remain. Here we review the challenges for GBM treatment, as well as the potential of unconventional T cells in cancer immunotherapy, with a focus on MAIT cells, their potential role in cancer, and their suitability as novel therapeutic targets in solid tumours such as GBM.

## Introduction: glioblastoma overview and challenges

Gliomas as a group account for over 80% of all malignant brain tumours and encompass both high-grade malignancies and low-grade tumours (1). Glioblastomas (GBMs) are classified by the World Health Organisation (WHO) as high grade (grade IV) gliomas. Recently, GBM nomenclature has been updated by the WHO to include molecular markers including isocitrate dehydrogenase (IDH) mutation status, categorising GBM tumours as IDH-wildtype or IDH-mutant (2). IDH mutations correlate with an improved chance survival of survival, however, a majority of GBM tumours are IDH-wildtype (3).

GBM is a relatively rare cancer, affecting approximately 7 individuals per 100,000 people in western populations (4, 5). However, GBM accounts for approximately half of malignant brain tumours, and exhibits disproportionately high mortality rates – the median survival time of 15 months from diagnosis (6), and a 5-year survival rate of 4.6%, which is markedly lower than other brain cancer types averaging 22.1% (5). Standard treatment for GBM, which typically combines surgical resection, radiotherapy and adjuvant chemotherapy (7), is largely ineffective at preventing recurrence, which is generally considered inevitable within 12 months. Overall, there is a clear need for more effective treatment options for GBM to improve patient outcomes and prevent tumour recurrence.

## Epidemiology and aetiology of GBM development

While no clear patterns of inherited susceptibility contribute substantially to GBM pathogenesis, several genetic risk factors have been identified. Oncogenic germline variants are estimated to be present in approximately 13% of individuals with GBM (8). Common mutations in GBM tumours include cyclin-dependent kinase inhibitor A2 (CDKN2A) and protein kinase B-Raf, which may be useful biomarkers to predict disease severity and treatment response (8, 9).

Regarding environmental contributions, ionising radiation is the only clearly established risk factor for GBM development, though the correlation is stronger in younger demographics (10). While some researchers report a correlation between mobile phone use and glioma development, a paucity of data prevents any robust conclusions being drawn (11, 12). Interestingly, a history of allergy, particularly respiratory allergies, correlates with a reduced risk of developing glioma, however no clear mechanism has been attributed to this correlation (13, 14).

### Tumour pathogenesis

GBM pathophysiology is both complex and variable, with tumours able to arise in any lobe of the brain parenchyma. GBM is characterised by invasive growth into normal brain tissue, whereby tumour cells interact with the extracellular matrix (ECM) (15, 16) and adjacent cells to degrade the ECM, facilitating spread of the tumour and simultaneously damaging brain tissue (17). This invasive pattern of growth into vital brain structures typically makes complete surgical resection impossible thus contributing to an extremely high rate of tumour recurrence (18).

Most GBM tumours harbour genetic and epigenetic alterations that disrupt cell-cycle control, drive over-expression of growth factors and promote rapid proliferation, angiogenesis, migration and invasion of surrounding tissue (19, 20). Mutations to the p53 gene (19, 21) are the most common in oncogenesis of GBM, with the gene being responsible for regulating normal cell proliferation (22). Loss of cell cycle control in GBM occurs due to loss of normal p53 function, as well as through disruption to the p16^INK4a^ pathway, both of which contribute to major cell proliferation checkpoints at which a cell commits to division (23–25). Alterations to the epidermal growth factor receptor (EGFR), and downstream signalling molecules phosphatidylinositol-3-OH kinase (PIK3) the negative regulator phosphatase and tensin homologue (PTEN), which are important for controlling cell proliferation and apoptosis, are frequent in GBM (23, 25). The potential to utilise these mutations as therapeutic targets in GBM has been reviewed elsewhere (26). It should be noted that after initial loss of cell cycle control, rapidly proliferating cells exhibit a high level of genetic, molecular and metabolic heterogeneity, making tumour cells a difficult target for local immune surveillance due to inherent variability (21, 27). Indeed, the degree of hypermutation is such that recurrent GBM tumours are often genetically unrecognisable from the initial tumour (28).

The formation and development of tumour tissue is highly dependent on the secretion of IL-6, which is derived from various sources but most notably from the tumour cells themselves (29) and tumour-associated macrophages (TAMs) (30). A vital step for tumour progression is the ‘angiogenic switch’, at which point tumour vasculature is established by gaining access to the normal tissue blood supply via disruption of blood vessel membranes and generation of intra-tumoral blood vessels (17, 31). Compared to most other solid tumours, GBM tumours are highly vascularised, which may allow for increased invasive growth (17, 32). Vascularisation is further driven by hypoxic conditions, which activate the hypoxia-induced factor 1 (HIF1)-vascular endothelial growth factor (VEGF) pathway, promoting endothelial cell proliferation and migration, thereby facilitating neovascularisation (27, 32). Tumour cells also undergo metabolic reprogramming to utilise aerobic glycolysis, characterised by rapid uptake of surrounding metabolites, in response to the hypoxic and a nutrient-poor tumour microenvironment (TME). This is triggered by HIF1a activation and NF-kB signalling, and subsequent upregulation of glucose transporter GLUT1 (33). In addition to hypoxia, this further destabilises surrounding healthy tissue due to poor nutrient availability (27). Ongoing hypoxia and necrosis in the tumour core result in damage to healthy brain tissue, leading to the accumulation of necrotic tissue mass and cell debris, as well as thrombosis in the tumour vasculature (27).

### Immune suppression in the GBM TME

A weak immune response to GBM is a major driver of poor clinical outcomes. Several factors allow GBM tumours to resist anti-tumour immunity, including restricted immune cell infiltration due to the blood brain barrier (BBB), a high level of tumour heterogeneity, and the immunosuppressive GBM TME (34, 35). GBM tumours are known as immunologically ‘cold’ tumours characterised by infiltration of immunosuppressive macrophages and myeloid cells, with poor lymphocyte infiltration (15, 36).

Under homeostatic conditions, the BBB prevents the transport of most molecules from the vasculature into the brain parenchyma (36–38). It is maintained by astrocytes, which cover the vasculature in the brain and maintain tight endothelial junctions to prevent unwanted transfer across the membrane (37). While the barrier can be partially disrupted in disease settings, including GBM (37), the BBB reduces the infiltration of tumour-targeted immune cells, effectively providing protection for tumour cells from immune detection (36, 38). Delivery of therapeutics across the BBB is also challenging, requiring specific barrier permeability enhancers that allow drug delivery without causing long-term damage (18, 39).

While small populations of tissue-resident T cells have been defined in the brain (40–42), generation of tumour-specific responses is hampered by the high level of tumour heterogeneity reducing the potential of tumour clearance in response to any single GBM antigen (21, 27, 43, 44). Additionally, tumour-infiltrating lymphocytes (TILs) can become exhausted or anergic in GBM and fail to respond to the tumour (43, 45). T cells isolated from individuals with GBM are poor producers of the T cell survival cytokine IL-2, and exhibit an exhausted phenotype characterised by expression of programmed cell death 1 (PD-1), T cell immunoglobulin and mucin domain-containing protein 3 (TIM-3), T cell immunoreceptor with Immunoglobulin and ITIM domains (TIGIT), and Lymphocyte-activation gene 3 (LAG-3) (46), indicating severe and possibly irreversible exhaustion (47). While recent approaches to treating tumours aim to target and reverse exhaustion in cytotoxic T cells, the upregulation of multiple checkpoints, as well as the terminal exhaustion phenotype, has impeded their success in GBM (34, 43, 47), thus necessitating alternative approaches.

T cell activation within the tumour is also impeded by tumour-derived signals suppressing activation of dendritic cells, thereby impairing tumour antigen presentation (48). These immunosuppressive signals include cell surface inhibitory proteins cytotoxic T-lymphocyte antigen 4 (CTLA-4) and PD-L1, which are expressed on GBM tumour cells and directly impair both dendritic cell and T cell activation (34, 44). Additionally, unusually high proportions of tolerance-inducing T_reg_ cells have been reported in GBM, reaching over a quarter of TILs in some cases (46, 49).

Myeloid cells including monocytes, macrophages and microglia are the most abundant population of tumour-infiltrating immune cells in GBM and contribute to the immunosuppressive TME in several ways (43). Myeloid-derived suppressor cells (MDSCs) are a phenotypically distinct population of neutrophils and monocytes, which exert potent immunosuppressive signals (50), and are associated with more advanced tumours and poor clinical outcomes in GBM (51). TAMs also represent a major subset of infiltrating immune cells, which lack of expression of co-stimulatory markers CD40 and CD86, required for initiation of T cell responses (43). In GBM, TAMs are clinically important as they are associated with poorer overall survival outcomes (52).

In addition to an immunosuppressive TME, systemic treatment-induced immunosuppression is common in individuals with GBM. Dexamethasone, a corticosteroid commonly used to attenuate the side effects of radiation therapy, has been strongly associated with systemic adverse effects and decreased overall survival in GBM (53, 54), likely due to the immunosuppressive properties of corticosteroids (55). There is also increasing evidence that corticosteroid use may interfere with current immunotherapies including checkpoint inhibition (53–55). Temozolomide, the most commonly used chemotherapy drug for GBM, is a powerful inhibitor of lymphocyte expansion, and systemic temozolomide may to decrease efficacy of anti-PD-1 treatment as it resulted in severe depletion of TILs in a pre-clinical GBM model (56). Moreover, dosage and timing of each individual therapeutic will be critical to the successful implementation of combination immunotherapy treatments as they progress into the clinic.

## Immunotherapies for GBM

The standard treatment protocol for GBM, named the ‘Stupp protocol’, involves maximal safe surgical resection (57) followed by radiotherapy and concurrent treatment with adjuvant chemotherapy, most commonly temozolomide (58–60). However, even in combining radiotherapy and chemotherapy after surgical resection there is only a modest increase in the 5-year survival rate to 11% (58, 59). Almost all individuals on this combination therapy experience recurrent tumour growth within 6–7 months (61). Bevacizumab, a monoclonal therapy targeting VEGF-A has been shown to extend progression-free survival, but not overall survival, and is approved as a second-line treatment for recurrent GBM (62–64). A recent trial demonstrated improved progression-free and overall survival for individuals receiving temozolomide in combination with Bevaciumab in individuals with high cyclooxygenase-2 (COX-2) expression (65). Given the overall poor prognosis, many treatments for individuals with GBM are instead focussed on symptomatic relief to promote quality of life, as disease progression can include serious comorbidities (66–69).

Advances in understanding of both cancer biology and cancer immunobiology have led to the development of several new immune-based therapeutic approaches for cancers, over the last decade. The most notable immunotherapy approaches gaining traction are monoclonal antibodies and more recently, cell-based therapies including chimeric antigen receptor T (CAR-T) cells (**Table 1**). However, the prohibitive immunological landscape and potential interactions with existing treatments have significantly hampered efficacy of these immunotherapies in GBM.

**Table 1:** Active or recruiting trials of GBM immunotherapies as of May 2026.

ClinicalTrials.gov ID	Phase	Therapeutic
Antibody therapies		
NCT05432518	Early phase I	Afatinib, Dasatinib, Palbociclib or Oloparib
NCT06917885	Phase I	HLA-peptide and IL-2 fusion protein
NCT06258018	Phase I/II	Niraparib
NCT06630260	Phase I/II	Avutometinib + Defactinib
NCT05734560	Phase I/II	D2C7-immunotoxin (anti-EGFR) *plus* 2141-V11 (anti-CD40)
NCT06336291	Phase II	L19-TNF (anti-fibronectin-TNF fusion protein)
NCT06816927	Phase II	Nivolumab + Relatlimab
NCT04559230	Phase II	Sacituzumab
NCT03970447	Master protocol adaptive phase II/III platform trial	Regorafenib or Paxalisib
NCT05235737	Phase IV	Neoadjuvant Pembrolizumab
Cell-based therapies		
NCT07343986	Phase I	Autologous anti-EGFR anti-tumour CAR T cells
NCT05353530	Phase I	Autologous IL-8 receptor modified CD70-specific CAR T cells
NCT04003649	Phase I	Autologous IL13Rα2-specific, 4-1BB co-stimulatory, CD19 truncated expressing CAR T cells
NCT04165941	Phase I	Autologous genetically modified chemotherapy resistant γδ T cells
NCT04991870	Phase I	Allogenic genetically modified TGFβR2- NR3C1- natural killer cells
Vaccination based therapies		
NCT04573140	Phase I	Autologous total tumour mRNA lipid nanoparticle vaccine
NCT06805305	Phase II	Tumour antigen pulsed dendritic cells + pegylated IFN

### Chimeric antigen receptor T cells

Autologous chimeric antigen receptor T (CAR-T) cell therapies utilise an individual’s own T cells, which are engineered *ex vivo* to express a receptor complex (CAR) targeted towards a marker expressed by the tumour cells, then re-infused into the patient (70). Multiple generations of CAR have been developed with more recent generation CARs including co-stimulatory molecules such as CD28 and 4-1BB (CD137) to enhance the T cell response (reviewed in (71)). To date, most CAR-T cell therapies have been developed for, and shown efficacy in, haematological cancers. Several CAR-T cell treatments have now been FDA-approved – for example Axicabtagene (Yescarta; Kite Pharma) (72), and Tisagenlecleucel (Kymriah; Novartis) (73), targeting CD19, the first of several such treatments to be made available in 2017 and 2018, respectively. CAR-T therapy does, however, present several limitations. Serious adverse events such as cytokine release syndrome and CAR-T cell related encephalopathy syndrome have hampered the success of some therapeutics in the clinic, particularly those that utilise CD28 or 4-1BB as a co-stimulatory factor. While these conditions often resolve without intervention, in some cases they have had severe clinical manifestations and can lead to death, highlighting the critical need to monitor toxicity (71).

Efficacy of CAR-T cell therapy in solid tumours is vastly reduced due to poor tumour infiltration, tumour heterogeneity, and immunosuppressive signals in the TME (74). Currently, there are four active trials evaluating CAR-T cells in GBM (**Table 1**) (75). Attempts to develop GBM-specific CAR-T cells have generated a treatment targeted towards epidermal growth factor receptor (EGFR) variant 3 (EGFRvIII) (76), a GBM-evaluation is ongoing (NCT07343986). Recently, Choi et al. treated three individuals with GBM using EGFRvIII-targeted CARs, observing tumour regression following intraventricular infusion of the CAR-T cells (77). However, in this trial the durability of the treatment was only sustained for greater than 150 days in one of the three enrolled individuals (77). Other approaches have targeted CARs to IL-13Rα2, a high-affinity IL-13 receptor. Brown et al. demonstrated that IL-13Rα2 CARs displayed transient anti-tumour activity (78), but ultimately patient outcomes did not improve, likely due to downregulation of the target receptor (79).

Lastly, targeting human epidermal growth factor receptor 2 (HER2), which is expressed by ~80% of GBMs (80) with CAR-T cells has shown promise *in vitro* (81), and while HER2 is also expressed in healthy tissue (82) a phase I clinical trial using HER2-engineered virus-specific T cells was well tolerated and showed clinical benefits in some patients (83). CAR-NK cells may have some advantages over CAR-T cells, offering the potential for “off-the-shelf” therapies (reviewed in (84)). For example, CAR-NK cells targeting HER2 (NK-92/5.28.z, derived from the NK-92 cell line), in combination with immune checkpoint blockade, have shown some promise in orthotopic mouse models of GBM, and are currently in Phase I trials (85). Overall, while initial results indicate CAR-T and CAR-NK cell therapies could be a promising avenue for new GBM therapeutics, further research is required.

### Immune checkpoint blockade

Exhausted T cells in individuals with cancer display increased expression of immune checkpoint markers – inhibitory regulators of activation – such as PD-1, CTLA-4 and TIM-3. Targeting these markers with monoclonal antibody therapies (immune checkpoint blockade) has proven effective in restoring T cell function in a range of cancers, reducing tumour burden and improving clinical outcomes. A range of FDA-approved checkpoint blockade therapies are in use including Ipilimumab and Tremelimumab targeting CTLA-4, and Nivolumab, Pembrolizumab and Cemiplimab targeting PD-1, for the treatment of melanoma, lymphoma and a range of other cancers (86). Further research is ongoing for other checkpoints, including TIM-3, VISTA and TIGIT (86). Combined therapies targeting more than one immune checkpoint have synergistic effects, for example, combination therapy of ipilimumab and nivolumab synergistically increased their efficacy in individuals with advanced melanoma (87).

Checkpoint blockade therapies have several limitations. The first is that some patients do not respond to treatment, most likely due to either an absence of antigen expression, genetic mutation to evade T cell responses, or an overwhelming immunosuppressive signal in the TME (88). Second is that adverse effects are common and discourage individuals from maintaining their treatment (89). While toxicity usually resolves, up to 10% of cases require medical intervention (89). Investigation into these mechanisms of therapeutic failure and adverse events, and the design of strategies to overcome these issues will be essential to improving treatment outcomes.

Clinical trials to date have assessed the efficacy of both intravenous and local delivery of immune checkpoint therapies as an addition to standard treatment in GBM. The anti-PD-1 monoclonal antibody Pembrolizumab has shown improvement in specific and systemic anti-tumour immune responses, evidenced by increased migration and infiltration of TILs, but when administered alone these did not translate to improved clinical outcomes (90, 91). A peptide inhibitor of CD200, another checkpoint implicated in GBM, showed some effect of overcoming immune tolerance induced by CD200 expression on tumour cells (92). A phase I trial assessing combined intracerebral delivery of combined αPD-1 and αCTLA-4 therapy showed good tolerance and promising clinical outcomes, although direct comparison of this approach with single-target therapies or standard treatment was not performed (93). Recently, neoadjuvant immune checkpoint inhibitor therapy, with administration of nivolumab (αPD-1), ipilimumab (αCTLA-4) and relatlimab (αLAG3) checkpoint inhibitors as a first line procedure prior to resection has shown promising results for one patient (94) and is currently in clinical trials for newly diagnosed GBM (**Table 1**). Collectively, new immunotherapy approaches still face significant challenges in GBM but present a promising approach to improve clinical outcomes for individuals with GBM.

## Unconventional T cells; a new promise for cancer immunotherapies

Unconventional T cells comprise several subsets including invariant natural killer T (iNKT) cells, γδ T cells, and mucosal-associated invariant T (MAIT) cells. These ‘innate-like’ T cells exhibit both innate and adaptive immune features that offer potential advantages to overcome the limitations of conventional T cell-based therapies. They have limited repertoires of T cell receptors (TCRs), which are restricted to antigen presenting molecules that, unlike the Major Histocompatibility Complex (MHC) Class I and II molecules, have limited polymorphism, thus reducing the likelihood of off-target reactivity. The rapid activation and effector functions of these cells are reminiscent of innate immune cells, as they can perform both cell signalling functions via cytokine release, and directly target antigen presenting cells with cytotoxic molecules (95). Thus, unconventional T cells are of interest for the development of both autologous and allogenic *‘off-the shelf’* therapies (96).

Invariant NKT (iNKT) cells use a semi-invariant αβ TCR comprising Vα14 joined to Jα18 TCRα chain, paired with a limited range of TCRβ chains in mice, and Vα24-Jα18 TCRα chain paired with a Vαβ11 TCRβ chain in humans. These TCRs allow recognition of lipid antigens produced by microbes, presented by the MHC class I-like molecule CD1d (97). iNKT cells display innate-like functions, rapidly producing a high level of cytotoxic molecules, including perforin and granzymes, upon antigen recognition. They are also capable of performing functions similar to T helper cell subsets; NKT1 cells have been shown to perform similar immune responses as Th1 cells, with other subsets releasing cytokines to recruit other immune cells including IL-17A (NKT17 cells), as well as anti-inflammatory IL-4 and IL-10 (NKT2) (98).

Invariant NKT cells are of interest in cancer immunotherapy due to their restricted antigen recognition and short *in vivo* lifespan, which together limit their potential for off-target toxicity. Development of iNKT-based immunotherapies has been conducted with mixed success. These therapies have shown great success in treating blood cancers, with a CD19 targeting allogenic CAR NKT cell therapy, showing up to a 43% complete response rate in trials of individuals with relapsed or refractory lymphoma (NCT03774654). In contrast, NKT cell therapies have shown only modest success in the treatment of solid cancers. A CAR-NKT cell therapy developed by Xu et al. in 2019 (99), showed limited success in phase I clinical trials, with injection of autologous NKT cells targeted to GD2 ganglioside – a molecule composed of glycosphingolipids linked with sialic acid chains, which is expressed by neuroblastoma cells – reducing metastatic bone lesions in 25% of neuroblastoma patients, and achieving complete response in 1 of 12 participants (100). In pre-clinical mouse models of orthotopic GBM, intra-tumoral injection of human stem-cell derived iNKT cells increased survival and decreased tumour volume (101). Similarly, CAR-NKT cells directed against EGFRvIII showed increased survival and decreased tumour volume in mice receiving human xenograft (102). Therefore, NKT cells present as a promising avenue for the development and refinement of both autologous and off-the-shelf allogenic therapeutic strategies, especially in seeking to treat cancers with unmet clinical need.

Gamma delta T cells constitute up to 5% of circulating T cells in humans (103). They use a limited number of TCRγ and δ chains, and exhibit reactivity to phosphoantigens (103). Populations of γδ T cells have been identified in barrier tissues, where their potent cytotoxic response and production of interferon (IFN)-γ confers protection against infection (104). γδ T cells have also generated interest in cancer research, due to their independence from MHC, their ability to recognise and lyse tumour cell lines in a TCR-dependent manner *in vitro* and demonstrated capacity to negatively regulate growth of tumours (105–107). A recent study characterised γδ T cells in GBM samples from 40 individuals, finding that γδ T cells could recognise and lyse autologous tumour tissue, while highlighting heterogeneity in the potency of responses between individuals (108). However, some studies have shown that a subset of IL-17 producing cells γδ T cells may promote tumour growth, creating ambiguity as to whether their role is protective or pathogenic (105, 109). While very few γδ T cell products have reached clinical trials, the development and potential of γδ CAR T cells as a therapeutic avenue for cancer treatments remains of interest and has been recently reviewed (110, 111).

## Mucosal-associated invariant T cells

The MHC class I-related protein (MR1) antigen presenting molecule has limited polymorphism, is highly conserved throughout evolution, and can be expressed by all nucleated cell types (112, 113). The best described and most abundant subset of MR1-reactive T (MR1T) cells in humans are MAIT cells, an innate-like subset of unconventional T cells, which use a semi-invariant TCRα chain comprised of the TCRα gene segment TRAV1-2 (also referred to as Vα7.2) joined to TRAJ33, TRAJ12 or TRAJ20, paired with a limited number of TCRβ chains (114). MAIT cells recognise metabolite antigens presented by MR1. The most potent MAIT antigens derive from the vitamin B2 synthesis pathway, which is essential for the metabolism of many bacteria and fungi. 5-(2-oxopropylideneamino)-6-D-ribitylaminouracil (5-OP-RU), the most potent known MAIT antigen, is formed by condensation of the riboflavin biosynthesis precursor 5-amino-6-D-ribitylaminouracil (5-A-RU) with the ubiquitous small metabolite methylglyoxal (115, 116).

In addition to 5-OP-RU, MR1 is known to accommodate molecularly diverse compounds including folates (6-formyl pterin; 6-FP, acetyl-6-FP) (116), drugs and drug derivatives (*e.g.*, diclofenac, and aspirin analogue 3-formylsalicylic acid) (117), sulphated bile acids (118), and vitamin B6 form pyridoxal (119), which are either antigens and non-agonistic/inhibitory compounds. While limited, the diversity in both TCRα and β chains used by MAIT cells can impact MR1-Ag recognition (120–122). Recent studies have also identified much smaller subsets of non-MAIT “MR1-reactive T cells (MR1T cells)” in human blood, that exhibit a more diverse TCR repertoire, and potentially heterogeneous effector functions (123). Some MR1T cells phenotypically resemble MAIT cells with high expression of CD161, IL-18R and promyelocytic leukaemia zinc finger (PLZF) (124, 125), and some TRAV1-2^+^ cells are not reactive to MR1-5-OP-RU tetramers but bind to 6-FP, microbial antigens, or are MR1 autoreactive T cells (123). Lastly, there are small numbers of TRAV1-2^-^ T cells that bind MR1-5-OP-RU tetramers, albeit with varying degrees of affinity (124). Notably, residual populations of MR1-5-OP-RU reactive cells also exist in *Traj33*^–/–^ mice, and like canonical TRAJ33^+^ MAIT cells (126), were shown to expand in response to bacterial infection (125). Recently, MR1T cells have been identified that appear to recognise endogenous or altered-self antigens and thus may be relevant to tumour cell immune recognition (127–129). MR1T cell clone MC.7.G5 by Crowther et al. was initially reported to recognise cancer cells via an unknown antigen and exert cytotoxic effector functions against a broad range of cancer cells, but not healthy cells, in an MR1-dependent manner (130). However, the specificity of this clone for tumour cells was challenged in subsequent studies (131). While their study is difficult given their low abundance in humans, identification of antigens is just emerging, and specific tools to allow accurate study of this population are yet to be developed, there is much interest in the potential recognition of tumour cells by non-MAIT MR1T cells.

The strong tissue-homing characteristics of MAIT cells may provide benefits for solid or non-circulating tumour applications, while the greater abundance of MAIT cells compared to iNKT could offer further advantages in terms of isolation and scale-up for clinical development. The anti-tumour properties of MAIT cells have generated strong interest despite a limited body of work to confirm their role in cytolysis of tumour cells, reviewed previously (95, 132). While currently there are few MAIT-targeted immunotherapies in development, MAIT cells represent a physiologically important and highly promising candidate for further development in this area.

### MAIT cells in infections and non-infectious pathologies

MAIT cells undergo TCR-mediated activation following recognition of MR1 bound metabolite antigens, with activation also dependent on co-stimulatory signals (126, 133, 134) and cytokines (134, 135). TCR-activated human MAIT cells rapidly secrete cytokines such as IFN-γ and tumour necrosis factor (TNF) (133, 136) and can lyse cells infected with bacteria (137, 138). MAIT cells play an important role in defence against bacterial infection as most clearly demonstrated in mouse models (reviewed elsewhere (139)). Conversely, they can drive pathology in chronic *Helicobacter pylori* infection (140) and in chronic sinus inflammation (141).

MAIT cells are also implicated in control of viral infections, including influenza (142, 143) and COVID-19 (144, 145). While viruses cannot produce riboflavin-based antigens, MAIT cells appear to be activated during viral infections in a TCR-independent manner by cytokines (143, 146) with their response slower and more limited than TCR-mediated activation (147). In patients with severe viral infections MAIT cells are reduced in number and functionally impaired (143, 146, 148, 149), and restoring their function may be of therapeutic interest (148).

A growing body of evidence implicates MAIT cells in immune-mediated pathologies including inflammatory bowel diseases, autoimmune diseases, and asthma and allergies (reviewed in (150)). MAIT cells also possess tissue repair functions (151, 152) and can accelerate wound healing in mice (153, 154). Furthermore, a role in maintenance of barrier homeostasis is now considered an important MAIT cell function (155), including an intriguing association with maintenance of meningeal barrier integrity (156).

### Mucosal-associated invariant T cell distribution and phenotype in humans and mice

MR1-5-OP-RU tetramers (115, 122) are now the gold standard reagents to specifically identify and isolate MAIT cells and have greatly improved accuracy of MAIT cell studies. Prior to their development, studies relied on surrogate markers such as CD3, TRAV1–2 and CD161, which are also expressed by other T cells and, in the case of CD161, not expressed at all stages of MAIT cell development (114, 122), and do not match MR1-tetramer reactivity, particularly in tissues (157). Notably, no surrogate markers for MAIT cell exist in mice. TCR transgenic, *Mr1*^–/–^ and *Traj33*^–/–^ mouse lines have been developed (125, 158, 159). These, along with methods for sterile antigen-dependent expansion of MAIT cells *in vivo* (126, 135, 160, 161), and classical tools such as adoptive transfer of MAIT cells, have allowed the assessment of MAIT cells in various infection and disease contexts.

While MAIT cells comprise on average 3-5% of total circulating αβ T cells in human blood (162), they are also found in both mucosal and non-mucosal sites including the lung, intestines, stomach, oral mucosa, genital tract, skin, thymus, liver, kidneys, lymphoid tissue, and central nervous system (114, 163). While circulating MAIT cell frequencies peak in early adulthood and decline with age (162), populations of tissue-resident MAIT cells remain relatively stable over the lifespan of individuals (164).

**Figure 1:**
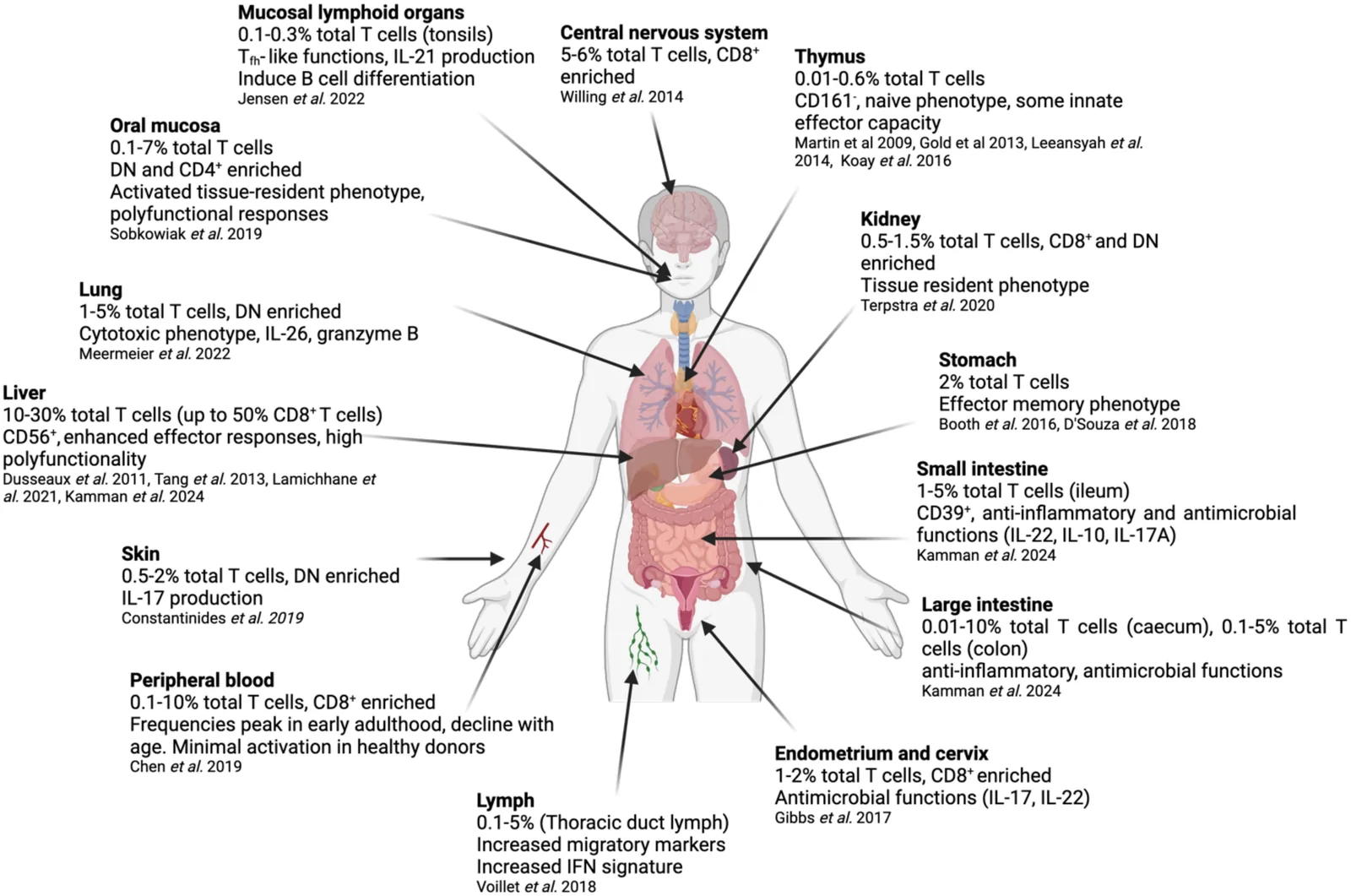
Distribution and phenotype of MAIT cells in humans. Frequencies and phenotypic traits of MAIT cells in healthy human tissues, as reported in the cited studies. Studies have defined MAIT cells as Vα7.2^+^ CD161^hi^ T cells or as MR1-5-OP-RU tetramer reactive T cells. Figure created with BioRender (235).

Most studies of human MAIT cells in disease have analysed circulating MAIT cells, but they also exhibit tissue-specific phenotypes (**Figure 1**). For example, MAIT cells residing in the liver are predominately CD8^+^, express the NK cell marker CD56 and display effector responses with a high level of polyfunctionality (164–167). In other tissues, such as the intestines (164), more potent anti-inflammatory and anti-microbial activity is observed, while a phenotypic bias towards IL-17 and IL-22 production is seen in MAIT cells residing in the cervix and endometrial tissue (168).

MAIT cells in laboratory mice are rarer than in humans but are nonetheless found at similar tissues site (139, 169). Both C57BL/6 and BALB/c mouse strains maintain populations of MAIT cells in the thymus, lymph nodes, liver and lungs; with notably higher MAIT cell frequencies in C57BL/6 mice (169). In most cases, frequencies are lower than the corresponding human tissue, for example in the lungs (126, 161, 170), liver (171, 172), and circulation (169). However, some tissues have higher frequencies in mice, for example in the skin, where MAIT cells comprise up to 40% of T cells compared to 2% in humans (154).

### MAIT cells in the brain

The first observation hinting at the presence of MAIT cells in the brain was in 2008, when Peterfalvi and colleagues reported Vα7.2 transcripts in biopsies of brain tumours (173). Subsequent studies have identified MAIT cells in the mouse brain and meninges (42, 156, 174), and showed that astrocytes and microglia can present antigen via MR1 to induce TCR-dependent MAIT cell activation (174). These findings collectively indicate a potential for MAIT cell activation and function within the brain, potentially reducing the limitations to immune therapy imposed by the BBB. Zhang et al. demonstrated that meningeal MAIT cells express antioxidant molecules and play a role in the homeostatic maintenance of meningeal barrier integrity, which was found to be greatly reduced in the MAIT-deficient *Mr1*^–/–^ mouse strain (156). Interestingly, adoptive transfer of MAIT cells into *Mr1*^–/–^ mice restored homeostatic and anti-inflammatory functions (156), suggesting that MAIT contributions to maintaining barrier function may be independent of MR1. In contrast, in a Vietnamese cohort, an *MR1* intronic polymorphism affecting expression was strongly associated with the development of meningeal tuberculosis (175). Thus, the role of MR1 in driving MAIT cell immunity in the brain may be context dependent.

Despite the identification of MAIT cells in brain tissue, few studies have investigated MAIT cells in the context of diseases affecting the brain and central nervous system. In individuals with multiple sclerosis (MS), MAIT cells decrease in the circulation but are detected in the cerebrospinal fluid and white matter lesions (176–180), suggesting their migration towards MS lesions. Circulating MAIT cells in individuals with MS exhibit a pro-inflammatory profile including production of IFN-γ and IL-17 (177, 178). Importantly, MS lesions were found to over-express MR1 and displayed elevated IL-18 and IL-23, congruent with a pro-inflammatory environment capable of eliciting TCR-dependent MAIT cell activation (179). Elevated expression *of MR1* was reported in the brain of people with Alzheimer’s disease (AD), and the accumulation of amyloid-beta plaques was reduced in the absence of MR1 in a the 5XFAD mouse model (181), suggesting a possible role for MAIT cells in AD. Circulating MAIT cells displayed an activated and exhausted phenotype with reduced IFN-γ producing capacity in people with Parkinson’s disease (182). Mouse models of ischaemic stroke indicated a reduction in infarct volume and improved recovery from neurological impairment in *Mr1*^–/–^ mice or mice treated with the MR1 ligand 6-FP, which competitively inhibits MAIT cell activation, hinting that neurological damage may be exacerbated by activated MAIT cells (183). Finally, high expression of *MR1* has been reported in both high- and low-grade gliomas and is associated with poor clinical outcomes (42, 184). Intriguingly, activated MAIT cells appear to be protective in glioma, but the role of MR1-mediated MAIT cell activation in this setting is unclear (42). Collectively, these studies suggest that MAIT cells may be important for maintaining homeostatic functions in a healthy brain, while activated and inflammatory MAIT cells can contribute to either immune protection or drive pathology in disease states.

## MAIT cells in cancer

Over the past decade, MAIT cells have been examined in several cancer types, with most studies focussed on mucosal cancers (185–189). However, current research has not reached consensus as to whether MAIT cells potentiate protective or pathogenic effector functions in tumour development, the factors that drive these divergent roles and how they may be harnessed for therapeutic benefit.

### Observations from human studies

Studies of mucosal cancers of the colon mostly demonstrate a decrease in the frequency of circulating MAIT cells in people with cancer compared to healthy participants (185–187), with MAIT cells exhibiting an increase in expression of migratory markers and a higher overall abundance compared to adjacent healthy tissue (186, 188, 189). However, this trend is not recapitulated across all cancer types. For example, patients with hepatocellular carcinoma were found to have both reduced circulating MAIT cells compared to healthy donors, and a lower percentage of MAIT cells in tumour tissue than healthy livers (187).

In several studies, an increase in intra-tumoral MAIT cell abundance was found to correlate with poor patient prognosis (187, 189–191). Large-scale transcriptomic analysis of a range of cancers by Yao et al. revealed either no correlation or a negative correlation between MAIT numbers and clinical outcome in a range of cancer types (192). In contrast, a higher frequency of MAIT cells has been found to correlate with improved clinical outcomes in several cancers, particularly blood cancers (193–195). In some cases, MAIT cells have been observed at higher frequencies in the early stages of tumour development but decline as the tumour progresses (196–198).

Circulating MAIT cells isolated from people with a range of cancers are functionally exhausted (**Table 2**), having impaired production of IFN-γ and TNF, and increased expression of exhaustion marker CD39 (199) alongside immune checkpoint molecules PD-1, CTLA-4 and TIM-3 (185, 187). These findings indicate susceptibility of MAIT cells to immunosuppressive signals in the TME, which prevent anti-tumour functions, and which may contribute to the correlations with poor prognosis in some cancers (191, 200, 201). *In lieu* of functional T_h_1-like responses, MAIT cells have been reported to instead demonstrate T_h_17-like or immunoregulatory roles in cancer. Indeed, populations of CD4^+^ FoxP3^+^ MAIT cells have been described in samples collected from patients with colorectal cancer and hepatocellular carcinoma, and these cells have further been shown to suppress anti-tumour functions of CD8^+^ T cells, and thus drive tumour progression (188, 199). MAIT cells can produce IL-17 to as an effector response, but whether IL-17 plays protective or pathogenic role in tumorigenesis remains unclear. The extent of IL-17 production is variable across cancer types, with some studies observing increased IL-17 production (185, 196, 200), and others showing decreased IL-17 production (188, 195, 202). Findings by Ling et al. suggest that elevated IL-17 contributes to MR1-driven tumour suppression in colorectal cancer (185), but others indicate that skewing towards an IL-17 dominant response contributes to tumorigenesis (200). Overall, these studies suggest nuanced roles for MAIT cells between different tumour types, likely driven by signals specific to the tissue of origin and TME.

**Table 2:** Analysis of MAIT cells in clinical samples from people with cancer.

Cancer type	MAIT cell source	MAIT populations	MAIT cell activation	Association with clinical outcomes	Other observations	References
Acute Myeloid Leukaemia	Blood and bone marrow	Circulating MAITs ↓	Immune checkpoints ↑ IL-17 ↑ IFN-γ and TNF ↓	Higher MAIT frequency correlated with improved prognosis	MAIT frequency ↓ during chemotherapy, recovered post-treatment.	(194, 228)
Breast cancer	Tumour samples	Tumour-infiltrating MAITs detected	IL-17 ↑	Unknown	MR1-dependent activation in response to tumour cells.	(229)
Cervical cancer	Blood samples	Circulating MAITs ↓		Fewer circulating MAIT cells correlated with disease progression.		(198)
Colorectal cancer	Blood and tumour tissue	Circulating MAIT cells reported as ↑ or ↓ Tumour-infiltrating MAIT cells detected. MAIT proliferation ↑ Migratory markers↑	Type I responses ↓ or maintained. IL-17 ↓ Exhaustion ↑ Activation enhanced by bacterial infiltration into tumours. Subset of CD4^+^ FoxP3^+^ MAIT cells detected	MAIT cells induced tumour cell cycle arrest. Higher MAIT frequency associated with poor prognosis.	Cell cycle arrest abrogated by anti-MR1. Effector function can be restored by anti-PD-1.	(185, 186, 189, 190, 199, 230, 231)
Hepatocellular carcinoma	Blood and liver tissue	Circulating and liver resident MAIT cells ↓ Migratory marker CXCR3^+^ ↑	Activation ↓ Exhaustion ↑ Immunoregulatory functions	Increased MAITs associated with poor prognosis.		(187, 191, 232)
Melanoma	Blood and tumour tissue	Circulating MAITs ↓ Tumour-infiltrating MAIT cells detected.	Activation ↓ PD-1 ↑	Higher MAIT cell frequencies correlated with successful anti-PD-1 treatment and improved survival outcomes.	Circulating MAIT populations restored by anti-PD-1.	(193, 203)
Metastatic colorectal cancer (Hepatic)	Peripheral blood, healthy liver and tumour tissue	Tumour infiltrating MAITs ↓ compared to healthy liver	IFN-γ ↓ Effector response to MR1-dependent stimuli ↓ No change with chemotherapy.			(233)
Multiple myeloma	Blood and bone marrow samples	Circulating MAITs ↓	Impaired MAIT functionality. PD-1 ↑		Function restored with anti-PD-1 treatment	(204, 207)
Non-small cell lung cancer	Blood and tumour tissue	Circulating MAIT cells unchanged or ↓, Tumour-infiltrating MAIT cells detected Migratory markers ↑ CXCR6^+^ MAIT cells identified	Exhaustion markers ↑ IL-17 ↑	CD8^+^ MAIT and IFN-γ responses correlate with response to anti-PD-1 therapy. Higher proportion of MAIT17 type responses in non-responders	MAIT frequencies higher in stage I compared to stage IV tumours	(196, 200, 201)
Oesophageal cancer	Blood and tissue samples	Circulating MAIT cells ↓, tumour-infiltrating MAITs detected	Activation ↓ PD-1 ↑	MAIT cell frequency ↑ in patients with less advanced tumours		(197)
Ovarian cancer	Blood, ascites and tumour tissue	Tumour-infiltrating MAIT cells detected Migratory markers ↑	Activation and cytotoxic potential	Activated MAIT cells correlated with response to chemotherapy	Tumour-infiltrating MAITs capable of response to MR1 dependent and independent stimuli	(195, 202)
Prostate cancer	Blood samples	MAIT frequency unchanged compared to healthy controls	Response to *in vitro* stimuli ↓ PD-L1 and PD-L1 upregulation in response to stimuli ↑		MR1-dependent stimulation + anti-PD-1 therapy associated with tumour control *in vitro*	(205)
Renal carcinoma	Blood samples	MAIT frequency unchanged compared to healthy controls. CD4+ MAIT% ↑	Activation ↓ PD-1 ↑	Activation restored, exhaustion reversed by faecal microbiota transplant (FMT).	MAIT cells important in favourable response to FMT therapy.	(206)
T cell non-Hodgkin’s lymphoma	Lymphoma biopsies	MAIT cells detected in ~25% of lymphoma samples			Some variation in TCR clonality between patients.	(234)

Importantly, MAIT cells have been implicated in driving responses to several immune modulating treatments. Indeed, the abundance and activation state of MAIT cells has been correlated with responsiveness to anti PD-1 therapy in the context of both blood cancers and solid tumours (190, 193, 194, 200, 201, 203, 204). MAIT cell frequency has been proposed as a potential biomarker to predict responsiveness to anti-PD-1 therapy in melanoma patients (193, 203). Characterisation of functional subsets suggest that T_h_1-like functions, rather than T_h_17-like functions in tumour-infiltrating MAIT cells drive this correlation (201), and assessment of the effect of αPD-1 combined with MR1 ligands (for example, 5-A-RU plus methylglyoxal) demonstrate enhanced anti-tumour cytotoxicity by MAIT cells *in vitro*, although it’s important to note that this was demonstrated with circulating MAIT cells isolated from healthy donors, which may not represent functional capacity of MAIT cells in people with cancer (205). Characterisation of MAIT cell responses in renal carcinomas before and after faecal microbiota transplant showed restoration of markers of MAIT cell activation and effector functions, as well as reversal of exhaustion as indicated by PD-1 expression (206), suggesting MAIT cells respond favourably to faecal microbiota transplant therapy. Favreau et al. reported greater activation in MAIT cells from people with multiple myeloma following *in vitro* treatment of the PBMCs with anti-PD-1and iNKT activating antigen α-GalCer – also highlighting a potential synergistic relationship between MAIT and iNKT cells in this context (204). Collectively, these findings demonstrate important roles for MAIT cells in immune modulating cancer therapies, highlighting the possibility that they may be a useful therapeutic target. Clinical findings to date and the roles of MAIT cells in various tumour types are summarised in **Table 2**. How MAIT cells are affected, and how they impact the overall immune response to immune checkpoint inhibition warrants further investigation.

### Assessment of MAIT cell anti-tumour functions *in vitro*

*In vitro* studies of human MAIT cells from healthy donors have demonstrated cytotoxic capacity against various tumour cell lines (**Table 3**). In these studies, MAIT cells were pre-activated and expanded with 5-OP-RU and pro-inflammatory cytokines (207), PMA and ionomycin (186) or IL-12 and IL-18 (197). Cytotoxicity of MAIT cells against GBM tumour cells has been reported, albeit these results follow extensive expansion with MR1-5-OP-RU tetramer conjugated to anti-CD28 beads, and co-culture of tumour cell lines with fixed *Escherichia coli* (208). MAIT cells isolated from healthy liver tissue also demonstrate cytotoxicity against K562 cells that is further enhanced by IL-15 (209). However, few studies have detailed the cytotoxic function of naïve tissue-resident MAIT cells, due to their scarcity and the complexity of these models. Further work is needed to examine the *in vivo* phenotypes and effector functions of MAIT cells in the TME, especially to understand the underlying mechanisms driving MAIT activation in this context and establish whether such activation is dependent on MR1.

**Table 3:** Evidence of MAIT anti-tumour cytotoxicity *in vitro*.

MAIT cell origin	MAIT cell stimulation	Target cell	Time period for killing	MR1 dependence	Reference
Healthy donor blood	PMA/ionomycin during coculture	K562 leukaemia cell line	4 hours	Not tested	(186)
Healthy donor blood	21-day expansion with 5-OP-RU, IL-2, IL-7, IL-12, IL-15, IL-18	RPMI-8226 and U266 (multiple myeloma cell lines)	20 hours	MR1 dependent	(207)
Healthy donor blood	TCR stimulation beads, IL-12 and IL-18	OE33 oesophageal cancer cell line	4 hours	Not tested	(197)
Healthy donor liver tissue	48-hour incubation with IL-15	K562 leukaemia cell line	12 hours	MR1 independent	(209)
Healthy donor blood	21-day expansion with IL-2, 5-OP-RU-MR1 tetramer/CD28 beads	T98G GBM cell line	12 hours	Partially MR1-dependent	(208)
Healthy donor blood	5-A-RU + methylglyoxal, anti-PD-1	PC3 prostate cancer cell line	> 48 hours	Inconclusive	(205)

### Findings from *in vivo* models

**Figure 2:**
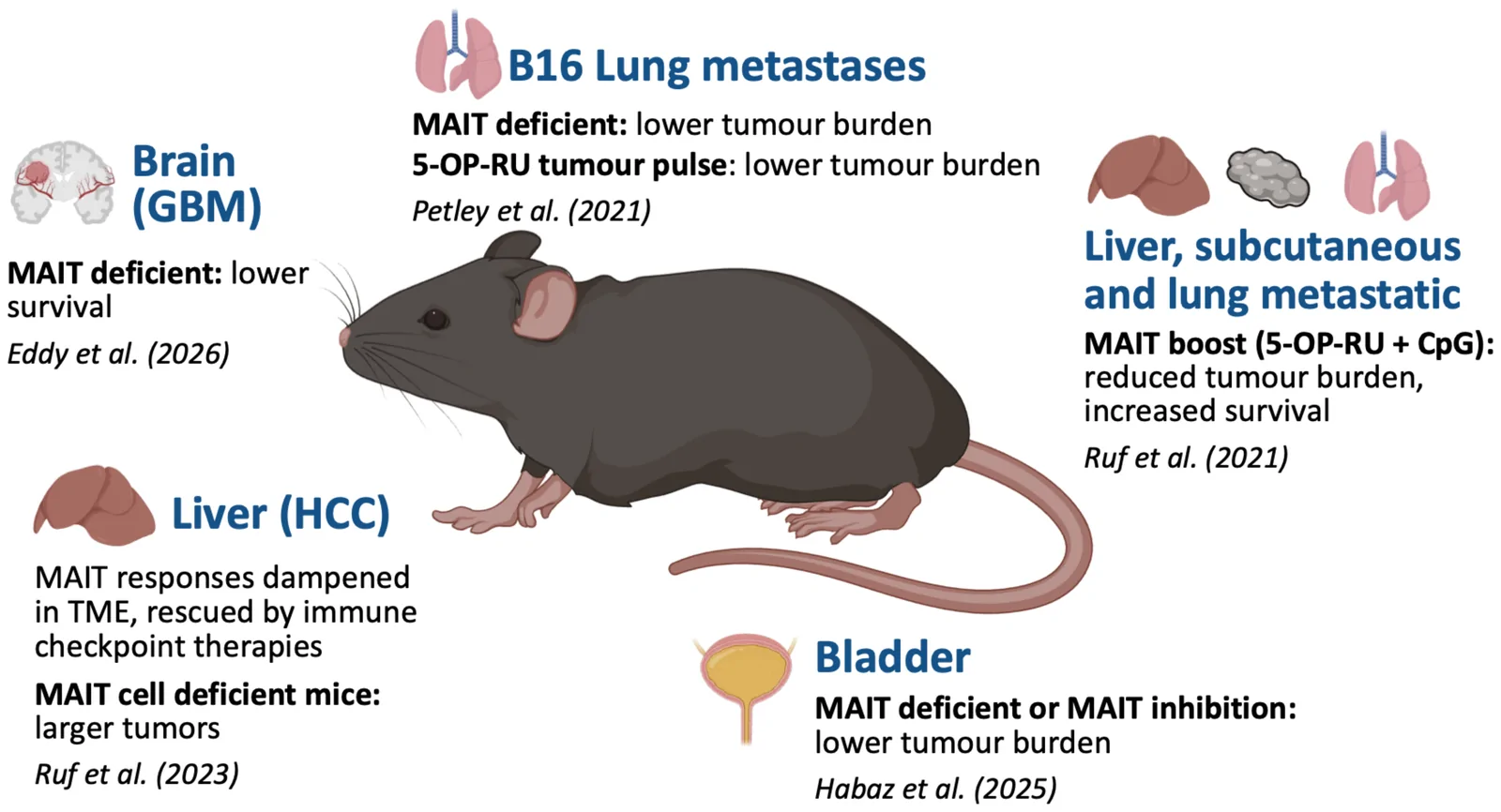
Impact of MAIT cells in mouse tumour models. A role for MAIT cells in tumour immunity has been demonstrated by comparing wild-type and MAIT cell deficient (*Mr1*^–/–^) mice, and by boosting MAIT cells or treating tumour cells with MAIT antigens. Results summarised from (42, 210–213). Image created with BioRender.

To date, few published studies have examined MAIT cell functions in murine tumour models (**Figure 2**). Petley and colleagues demonstrated that MAIT cell deficient *Mr1*^–/–^ mice showed reduced tumour burdens compared to WT mice, in a model of B16.F10 metastatic melanoma (210), suggesting MAIT cells are detrimental in this model. However, the same study showed reduced metastasis by pulsing the B16.F10 tumour cells with 5-OP-RU prior to intravenous injection (210), indicating a protective effect of MAIT cell activation. In another study, the activation and boosting of MAIT cells *in vivo* with CpG and 5-OP-RU also conferred a protective effect in reducing tumour burden and increasing survival in models of intrahepatic, subcutaneous and metastatic lung tumours (211). Systemic MAIT cell boosting with 5-OP-RU and adjuvants was also shown to increase MAIT cell abundance and modulate their function in the brain (42), but whether 5-OP-RU directly penetrates the brain to locally expand cells was not investigated in this study. MAIT cell cytotoxic effector functions and cytokine secretion were dampened in response to tumour growth and interactions with tumour-associated macrophages (TAMs) in models of hepatocellular carcinoma (212). However, these were somewhat rescued following treatment with immune checkpoint therapies (212). MAIT cell deficient mice had larger tumours in this study, suggesting a role in controlling tumour growth. In contrast MAIT cell deficient mice, or mice treated with acetyl-6-FP which can inhibit MAIT cell activation, showed lower tumour burdens in a model of bladder cancer, suggesting a detrimental role in combatting tumour growth (213). Taken together, potential anti-tumour functions of MAIT cells may depend on their activation state and may require MR1-dependent activation.

## Potential of MAIT cells as immunotherapy targets

**Figure 3:**
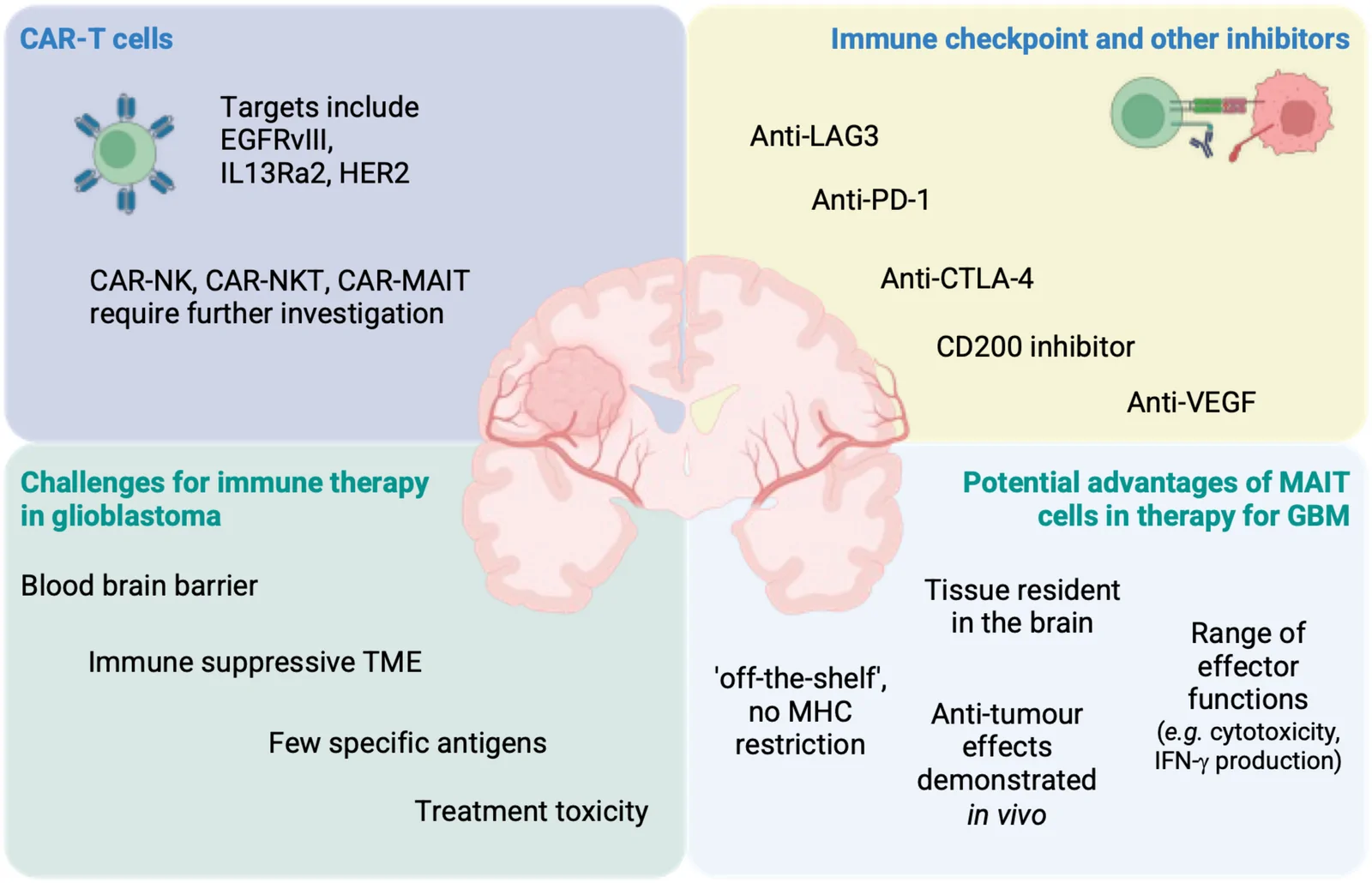
Summary of immune therapies, challenges in GBM and potential benefits of targeting MAIT cells. Image created with BioRender.

As development of cell-based cancer immunotherapies progresses, a strong interest in harnessing unconventional T cells for both autologous and off-the-shelf allogenic approaches has emerged (previously reviewed (95, 214, 215). Unconventional CAR-T approaches currently in clinical trials include NKT cells (216) and γδ T cells (**Table 1**). MAIT cells also present as an attractive candidate for immune cell-based therapies for several reasons (**Figure 3**). MAIT cells are relatively abundant T cells with rapid effector functions including cytotoxicity that also been successfully targeted towards tumour cells *in vitro* (186, 207, 209). Their restriction to the almost monomorphic MR1 protein due to their semi-invariant TCRs limits the variability of antigen recognition, and suggest a more predictable response can be achieved, as well as reducing the risk of alloreactive immune responses, allowing development of ‘off-the-shelf’ therapies that may negate the need for developing personalised treatment for each patient (95).

Detailed functional analyses of MAIT cells, including their capacity to restrain tumour growth or reduce tumour volumes, requires *in vivo* models. While the low abundance of MAIT cells in wild-type mice presents a challenge for such studies, investigative tools including MAIT-deficient knockout mouse strains (158, 217), MAIT cell-enriched mouse strains (218, 219) and models to boost MAIT abundance via vaccination (126), assist in establishing robust pre-clinical models to study MAIT cell function in cancer settings.

Initial evidence of MAIT cell infiltration into brain tumours was described by Peterfalvi and colleagues, who identified populations of tumour-infiltrating MAIT cells following detection of Vα7.2-Jα33 transcripts in GBM patient tumour samples, which were negative for NK cell marker CD56 (173), implying that tumour-infiltrating MAIT cells may not have a cytotoxic phenotype. Multi-omics analysis of the Cancer Genome Atlas (TCGA) revealed that high *MR1* expression can be observed relative to healthy individuals in both high- and low-grade gliomas, and *MR1* upregulation was associated with poor clinical outcomes, identifying a potential biomarker for predicting clinical outcomes (184), a finding since verified by further studies (42, 220). However, whether the correlation with poor overall survival is due to interactions between MAIT cells and MR1, which could influence MAIT cell activation state in the TME is less clear. As with other cancers, circulating MAIT cells were reduced in people with GBM compared to healthy controls (220). In GBM tumours, transcriptional signatures of MAIT cells were found to be associated with poor patient prognosis (220), but signatures of activated, rather than naïve, MAIT cells were associated with longer survival (42), suggesting therapies that activate MAIT cells may be beneficial. An *in vitro* study of MAIT-tumour interactions indicated that activated MAIT cells display partially MR1-dependent cytotoxic capacity against GBM cells (208), suggesting the potential of targeting MAIT cells therapeutically in GBM by harnessing existing cytotoxic functions. In the GL261 mouse model of GBM, *Mr1*^–/–^ mice, which lack MAIT cells, had reduced survival (42), suggesting a beneficial effect for MAIT cells in the context of brain tumours. In both humans and mice, MAIT cells in GBM displayed a Th17-like phenotype (42, 220), but the mechanisms behind MAIT cell protective effects in GBM remain unclear. Overall, more detailed characterisation of how MAIT cell phenotype and function is altered in the context of GBM could reveal a new therapeutic angle for disease treatment.

## Conclusions and remaining questions

While there is promise in exploring MAIT and other unconventional T cells as therapeutic targets in GBM, the methods by which these cells could be targeted in this setting is not yet clear. The *in vivo* phenotypes and effector functions of MAIT cells in the GBM TME first need to be defined, as well as the mechanisms (MR1-dependent or independent) driving MAIT cell activation. The potential direct anti-tumour functions of MAIT cells may depend on their activation through MR1. However, it is unclear if direct cytotoxic functions are required, and the complex interactions with other immune cells in the brain are yet to be examined.

While activated MAIT cells appear to be beneficial, MR1 expression is associated with worse outcomes. This apparent contradiction will be important to decipher in future studies. With the finding that MR1 expression is associated with poor survival, the inhibition or further modulation of MAIT cell activation through MR1, such as by the provision of non-agonist compounds [*e.g.*, 6-FP (116)] as has been performed in models of inflammatory bowel disease, metabolic disease and atopic dermatitis (221–223) should also be explored in pre-clinical models of GBM.

An important unanswered question is whether common GBM-associated genetic alterations influence MAIT cell biology through changes in tumour metabolism, MR1 ligand availability, antigen presentation, or the broader immune microenvironment. Defining these relationships may identify novel opportunities to incorporate MAIT cells into future precision immunotherapy strategies for GBM.

In a mouse model, MAIT cells were enriched to high numbers in the brain by systemic delivery of 5-OP-RU with adjuvants (42). Whether MAIT cells were expanded locally or trafficked from the periphery to the brain was not tested in this study. The small size of 5-OP-RU may suggest it could diffuse across the blood brain barrier. However, 5-OP-RU is chemically unstable, and thus, may require modification for use in therapeutic applications (224, 225). Formally testing the ability of MAIT antigens or antagonists to cross the BBB and understanding the ability of activated MAIT cells to enter the brain parenchyma, may be critical to understanding the therapeutic potential of MAIT cells in this setting.

In this light, it is considered that the tissue homing properties of MAIT cells, along with their cytotoxic capacity, may offer some benefits over conventional T cells in developing CAR-T therapies (CAR-MAIT) for solid tumours. The development of CAR-MAIT cells has not been reported but would be an interesting avenue to explore in GBM.

Finally, there is some evidence that MAIT cell abundance correlates with response to anti-PD-1 therapy in other cancers (193, 203), but this has not yet been explored in GBM. Other immune checkpoints such as CTLA-4 and LAG-3 should also be tested, as expression may differ between MAIT cells and other T cell subsets (226, 227). The mechanisms behind MAIT cell response to immune checkpoint inhibition warrant further investigation.

MAIT cells are relatively recently described and best known for their response to bacterial infections. However, the full scope of their contributions to immunity, including diverse functions in barrier integrity, anti-viral response and tumour immunity is rapidly emerging. Although many basic biological questions are yet to be addressed, their unique characteristics suggest they may provide benefits and new directions in tackling the challenges of GBM.
